# Protein Recognition of Linear and Cyclic Peptides of Homologous Sequences Implicated in the Aggregation of α‑Synuclein

**DOI:** 10.1021/acs.jpcb.5c05501

**Published:** 2025-10-13

**Authors:** Gabriel F. Martins, Cristiano Rocha, Nuno Galamba

**Affiliations:** BioISI-Biosystems and Integrative Sciences Institute, 111161 Faculty of Sciences of the University of Lisbon, C8, Campo Grande, 1749-016 Lisbon, Portugal

## Abstract

Various amino acid sequences have been suggested to play key roles in the aggregation of α-synuclein (α-syn), implicated in Parkinson’s disease and other synucleinopathies. A drug development strategy is, therefore, the design of molecules that bind to these sequences in the monomer. The latter, either alone or coupled with antiaggregation groups, could preclude homogeneous and/or heterogeneous primary nucleation by either blocking protein–protein interactions or stabilizing the monomer in its solution and/or membrane-bound conformations, respectively. Here, using molecular dynamics simulations, we assessed the specificity of *in trans* linear peptides (P1, NACore, and NACterm) and their cyclic counterparts toward homologous sequences in the N-terminal and NAC domains of α-syn, which have been experimentally shown to play key roles in aggregation. The results suggest that, despite some differences, both linear and cyclic peptides display specificity toward their homologous sequences in α-syn. Hence, these peptides have the potential to serve as recognition elements coupled with amyloid aggregation modulators or inhibitors. Additionally, most peptides stabilize the α-helices in the NAC region of α-syn when in a membrane-bond-like conformation and some induce more extended conformations when in a disordered form. However, our results also show that some peptides might eliminate intramolecular interactions with potential protective roles against aggregation. The results are further compared with the monomer at high temperatures, at which the protein adopts a more compact structure, and exhibits increased intramolecular β-sheet content, associated with an increase of the hydrophobic effect.

## Graphical abstract

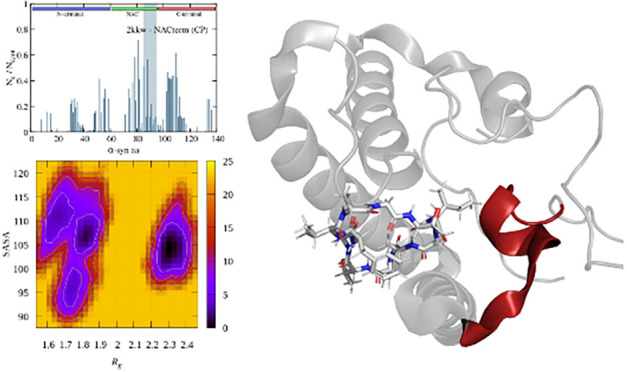

## 1 Introduction

The aggregation of α-synuclein (α-syn) into neurotoxic oligomers or protofibrils,^1–4^ and their accumulation in intracytoplasmic neuronal inclusions, called Lewy bodies and Lewy neurites,^5,6^ is linked to Parkinson’s disease (PD) and Lewy body dementia,^7,8^ which, together with multiple system atrophy are known as synucleinopathies.^9–13^

α-syn is a presynaptic 140 amino acid intrinsically disordered protein (IDP) mainly expressed in the central nervous system and found in soluble and membrane-bound forms.^14–19^ The protein consists of three domains: the N-terminal (N-term), a membrane-binding region encompassing amino acids 1–60 with a net charge of +5*e* that tends to form α-helices;^20^ the non-Aβ-amyloid component (NAC),^21^ an amyloidogenic segment comprising residues 61–95 with net charge −1; and the C-terminal (C-term), a disordered domain spanning residues 96–140 with a net charge of −13*e*.^14^

Whereas the reduced hydrophobicity and high net charge of α-syn (−9*e*) precludes the formation of persistent secondary and tertiary structures in the cytosol and water, hydrophobic interactions are thought to play a pivotal role to aggregation.^22,23^ The formation of parallel, in-register crossed β-sheets^24,25^ spanning the NAC (residues 61 to 95) and a neighbor segment of the N-term (residues 38 to 60) is a hallmark of these aggregates, whereas the C-term remains disordered and free.^26^ Furthermore, distinct polymorphs have been reported.^26–30^

Various studies identified amino acid sequences in the NAC^22,31–37^ and N-term^38,39^ domains that play key roles in the aggregation of α-syn.^23^ Thus, drug development strategies include the design of molecules that bind to these sequences in the monomer, blocking intermolecular protein–protein interactions (PPIs), and/or perturbing the conformational space of the monomer, potentially precluding misfolding and aggregation. In this sense, peptides are promising alternatives to small molecules as PPI-modifying drugs, mimicking protein surfaces and, therefore, competing for protein binding.^40–42^ Several peptides with the ability to inhibit the aggregation of α-syn were reported in the literature.^23^ Some of these are modified synthetic peptides of truncated segments believed to be pivotal to aggregation.^23,34,43–49^ Although the rationale behind this approach is that these peptides will compete for the same region in the protein, reducing or preventing aggregation, blocking these regions could also play the opposite effect, eliminating protective intramolecular interactions.^35,39,50,51^ Additionally, linear peptides suffer from some drawbacks, including proteolytic degradation and reduced membrane permeability. Macrocyclic peptides represent, in this sense, potential alternative drugs,^52^ holding the promise of enhanced specificity and potency, relative to small molecules, increased proteolytic resistance and membrane permeability,^53^ compared to linear peptides, and similar specificity to biologics such as monoclonal antibodies. However, perhaps more importantly, peptides have the potential to serve as recognition elements coupled with antiaggregation or aggregation modulator groups.^54^

Molecular dynamics (MD) simulations can, in principle, provide insight into linear and cyclic peptide selectivity and specificity for different regions of the target protein. However, whether this is observed in unbiased MD as well as the selectivity and specificity of cyclic peptides, compared to their linear precursors, remains largely unexplored. Here, we assessed whereas linear peptides and their cyclic counterparts bind to their homologous sequences in α-syn using all-atom ordinary MD simulations. Additionally, we assessed the effect of the addition of these peptides *in trans* to the conformational space of the α-syn monomer. We investigated two segments in the NAC region, namely, NACterm^35,55^ (residues _85_AGSIAAATGFV_95_) and NACore^31,36^ (residues _68_GAVVTGVTAVA_78_), and an N-term sequence, P1 ^38,39^ (residues _36_GVLYVGS_42_).

NACterm was shown to be involved in conformations of the monomer which could potentially inhibit aggregation.^35^ In particular, this sequence was found to interact with the sequence 110–130 from the C-term. Moreover, this segment comprises NAC’s longest hydrophobic sequence^23^ (_88_IAAA_91_) exhibiting a dimerization free energy similar to NACore.^56,57^ Rodriguez et al.^36^ and Bodles et al.^31^ showed that the latter formed neurotoxic amyloid-like fibrils, whereas a smaller segment (residues 68–76), although not forming fibrils, was the smallest peptide that still exhibited neurotoxicity.^31^ Doherty et al.^38^ showed that deletion or substitution of the pre-NAC sequence P1, prevents aggregation at physiological pH *in vitro*, and aggregation and toxicity in *C. elegans* models. Furthermore, P1 and P2 (residues 45–57) deletion was shown to prevent α-syn mediated vesicle fusion by altering the conformational properties of the protein when lipid bound.^38^ These motifs were, therefore, considered important both for the aggregation and the above putative function (i.e., regulation of neurotransmitter release)^58,59^ of α-syn. Various chaperones were also shown to recognize this motif, specifically, a segment around Tyr39, precluding aggregation.^60^ On the other hand, addition of a synthetic (capped) peptide with the sequence of P1 (with four residue extensions taken from the natural sequence of α-syn added to the N- and C-termini to enhance its solubility) (Ac-KTKE-_36_GVLYVGS_42_–KTKE-NH2) in equimolar or 10-fold molar excess, marginally increased the rate of fibril formation of wild-type (WT) α-syn.^39^ This was associated with the peptide binding to regions in the N-terminal (including P1 and P2) and NAC, further destabilizing protective^51^ intramolecular interactions between the N-term and/or NAC and the C-term.^39^

The main goal of this work is to determine whether cyclic peptides and their linear precursors recognize their homologous sequences *in cis* using standard molecular dynamics simulations, and to assess their influence on α-syn’s conformational space, including transient secondary structure transformations and/or the disruption of potentially protective intramolecular interactions.

## 2 Methods and Theory

Molecular dynamics (MD) simulations of the α-syn monomer modeled with CHARMM36m^61^ in 0.1 M NaCl aqueous solutions using the TIPS3P^61^ water model were performed in the isothermal–isobaric (*NpT*) ensemble in a cubic box with periodic boundary conditions, using the program GROMACS.^62^ The simulations were performed at 310 K and 0.1 MPa. The temperature (*T*) and pressure (*p*) were controlled with the Nosé-Hoover thermostat^63,64^ and the Parrinello–Rahman barostat,^65^ and the equations of motion were solved using the Verlet leapfrog algorithm with a 2 fs time-step. Electrostatic interactions were computed via the particle-mesh Ewald (PME) method.^66^ A cutoff of 1 nm was used for nonbonded van der Waals and for the PME real space electrostatic interactions. Heavy atom-hydrogen covalent bonds were constrained with the LINCS algorithm.^67^

The simulations were performed for two different starting conformers, specifically, the monomer in a fibril (PDB: 2n0a),^26^ and the monomer bound to a micelle of sodium lauroyl sarcosinate (PDB: 2kkw).^68^ The 2kkw structure is characteristic of a membrane-bonded α-syn conformation, in which the N-terminal and NAC domains form α-helical structures upon membrane binding. The 2n0a-based monomer adopted unfolded, although relatively compact, structures, whereas the latter (2kkw) exhibited persistent α-helical structure in the N-term and NAC regions even in the absence of a membrane, within the time scale of the simulations. Figure 1 shows representative conformations of α-syn found through clustering analysis. The latter was performed using the root-mean-square displacement (RMSD) clustering algorithm^69^ implemented in GROMACS with a cutoff of 0.5 nm. Additionally, MDs were carried out starting with these α-syn conformations and a single peptide (linear or cyclic) randomly inserted in the solvent. The following peptides were studied: NACterm^35,55^ (residues _85_AGSIAAATGFV_95_) and NACore^31,36^ (residues _68_GAVVTGVTAVA_78_), belonging to NAC, and an N-term sequence, P1 ^38^ (residues _36_GVLYVGS_42_). The peptides were cyclized through the addition of a covalent bond between the C- and N- termini. Figure 2 shows representative conformations of the linear and cyclic peptides. MDs of α-syn at 370 K and 0.1 MPa, in the absence of any peptide, were also performed to assess the effect of temperature in the conformational space of the monomer. The systems were equilibrated for 100 ns in the *NpT* ensemble following steepest descent energy minimization and a 500 ps equilibration period in the *NVT* ensemble. The trajectories were then propagated in the *NpT* ensemble for another 500 ns. All simulations were performed in 5 replicates. For α-syn in water, some trajectories were extended up to 1 μs to ensure sufficient sampling, allowing structural differences relative to the peptide solutions to be confidently attributed to protein–peptide interactions. Trajectory time-windows within the 500 ns, during which the peptides were bound to the protein were analyzed to probe the peptide’s influence on the protein structure. Additionally, analysis of the structure of α-syn was also carried out for the last 250 ns of each replicate to limit the potential memory of the starting conformation. Protein-peptide contact maps were assessed by averaging the number of contacts over the different MD replicas. A protein-peptide contact was defined by a distance *R* < 3.5 Å, where *R* is the minimum atomic distance between every atom of each residue of the protein and peptide. The secondary structure of the α-syn and peptides was assessed using the program DSSP (Dictionary of Secondary Structure of Proteins)^70^ which uses a strictly energetic hydrogen-bond definition (*E*_HB_ < −0.5 kcal/mol).

**Figure 1:**
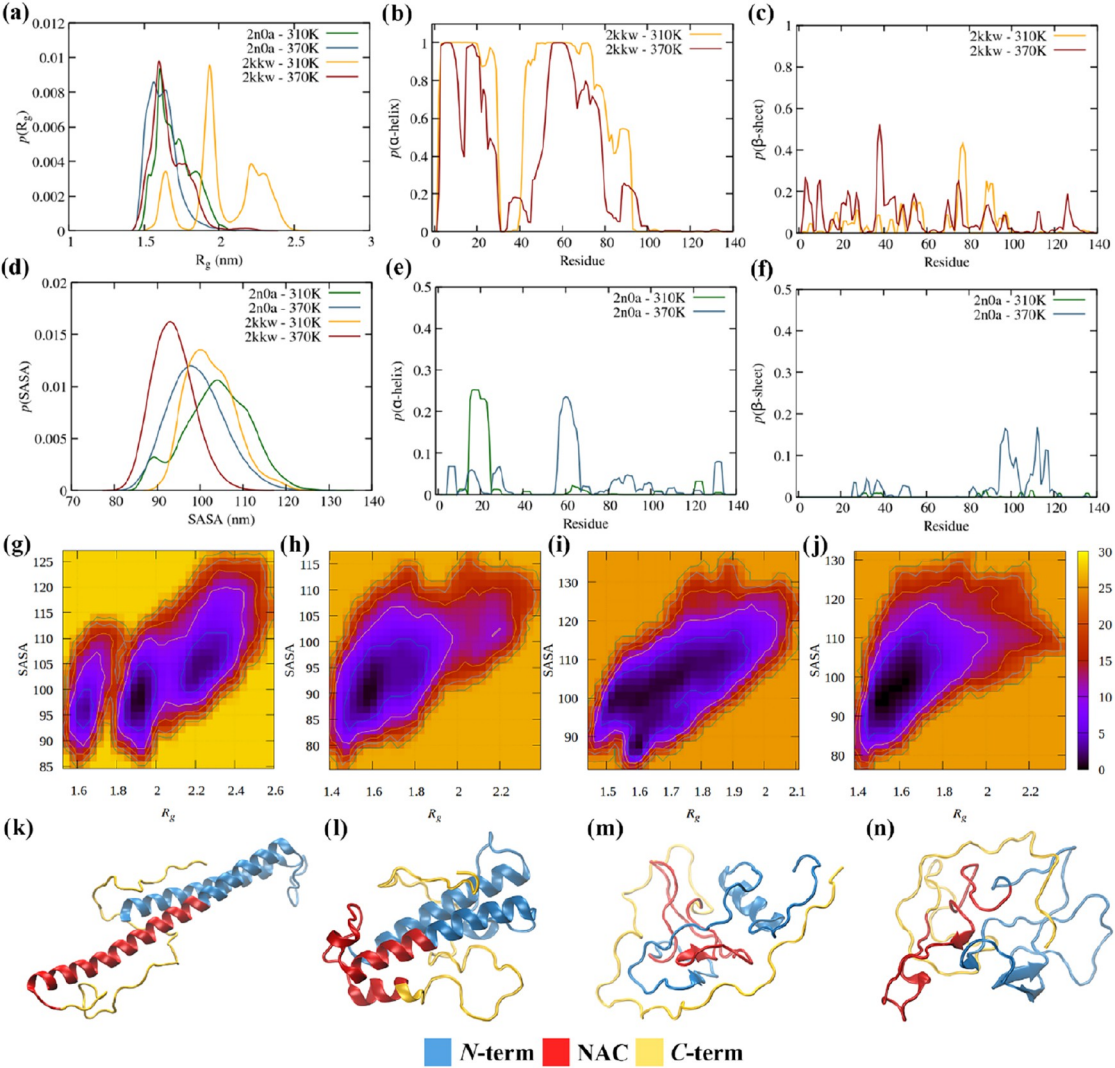
α-syn monomeric structure at 310 and 370 K, starting from fibril (2n0a) and micelle-bound (2kkw) conformations. (a) Distribution of the radius of gyration (*R*_g_); (b) distribution of α-helix structure per residue, p­(α-helix), for 2kkw; (c) distribution of β-sheet per residue, p­(β-sheet), for 2kkw; (d) distribution of the solvent accessible surface area, p­(SASA); (e) distribution of α-helix structure per residue, p­(α-helix), for 2n0a; (f) distribution of β-sheet per residue, p­(β-sheet), for 2n0a; α-syn reduced free energy surfaces (FES) in kJ/mol calculated using *R*_g_ and SASA for (g) 2kkw at 310 K, (h) 2kkw at 370 K, (i) 2n0a at 310 K, and (j) 2n0a at 370 K; (k) average structure from cluster analysis of α-syn at 310 K (2kkw); (l) average structure from cluster analysis of α-syn at 370 K (2kkw); (m) average structure from cluster analysis of α-syn at 310 K (2n0a); (n) average structure from cluster analysis of α-syn at 370 K (2n0a).

**Figure 2:**
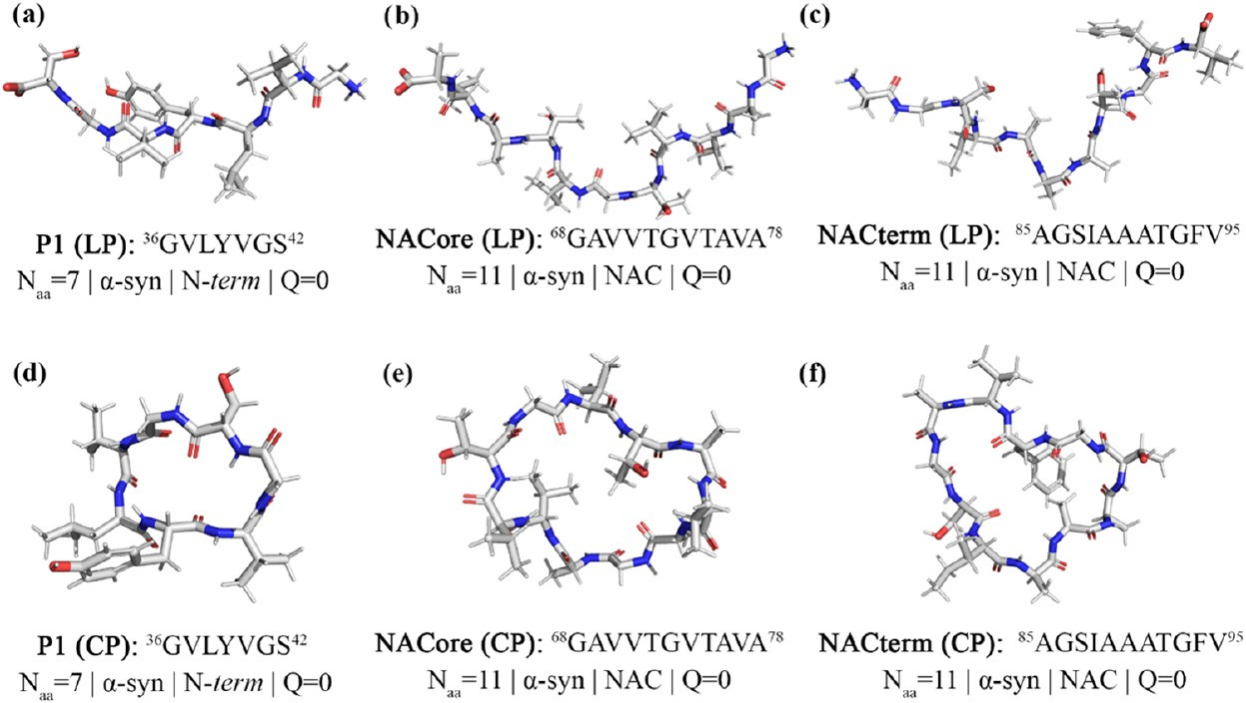
Average molecular structure determined by clustering analysis of the peptides studied. (a) P1 in the linear form (LP); (b) NACore in the linear form (LP); (c) NACterm in the linear form (LP); (d) P1 in the cyclic form (CP); (e) NACore in the cyclic form (CP); (f) NACterm in the cyclic form (CP).

## 3. Results and Discussion

A conformational transition of natively unfolded α-syn into a misfolded intermediate with increased β-sheet content, more aggregation-prone, is believed to precede aggregation,^25,71–73^ although the mechanisms and cellular environment underlying this structural transition to neurotoxic aggregates remains elusive. There is also evidence that primary nucleation might be induced by membrane interactions, triggering the conversion of α-syn from its soluble state into the aggregated state, significantly increasing the rate of primary nucleation.^74^ Another mechanism foresees a transformation of α-helical into a coiled structure upon α-syn’s penetration in the membrane, promoting the formation of oligomers in the membrane.^75^ In this sense a peptide that stabilizes the α-helical structure of the membrane-bound monomer could preclude oligomerization. To sample α-syn’s spatiotemporal conformational heterogeneity we analyzed the specificity of the linear and cyclic peptides and their impact on α-syn’s structure, starting from both conformations (see Section 2.). As mentioned in the Methods Section, the helical structure of the micelle-bond monomer (2kkw) persisted in aqueous solution in the absence of a membrane. Whereas this may or not be an artifact of the force field, it allowed assessing the peptides’ specificity toward this conformation in the absence of a membrane. Thus, our simulations probe the effect of the peptides on α-syn, while neglecting potential interactions with the membrane. Moreover, opposite to a membrane-α-syn system, our simulations allow for interactions with the less accessible N-terminal, the membrane binding domain of α-syn, which contains multiple KTKEGV repeats that drive helix formation.^14^

We assume that aggregation is triggered by specific intracellular modifications^51^ that reduce the solubility of WT α-syn enhancing hydrophobic interactions among monomers, driving homogeneous or heterogeneous^74^ (i.e., membrane promoted) primary nucleation. *In vitro*, α-syn aggregation is accelerated at high temperatures and lower pH.^72^ To gain insight into the effect of the hydrophobic effect enhancement in the monomeric structure we also simulated the monomer at 370 K. Hydrophobicity increases with the temperature^76–78^ and the monomer should, therefore, display a more compact structure by favoring intramolecular hydrophobic contacts and possibly the formation of secondary structure, namely, intramolecular β-sheets. These results are now discussed.

### 3.1. Temperature Effect

Figure 1 shows the distribution of the radius of gyration (*R*_g_), the solvent accessible surface area (SASA), and the α-helix and β-sheet (Figure 1a–f) for the α-syn monomer at 310 and 370 K, starting from the monomer in a fibril (2n0a) and a micelle-bound conformation (2kkw). Additionally, 2-dimensional free energy surfaces (Figure 1g–j) and the most probable structures of α-syn are displayed (Figure 1k–n).

A reduction of the conformational space at 370 K is observed for both starting conformations. Interestingly, this reduction is more noticeable for the 2kkw conformation, with *R*_g_ and SASA distributions displaying a more pronounced narrowing, and a shift to lower values, indicating more compact structures. This increase in compactness with temperature is consistent with single-molecule Förster resonance energy transfer experiments for several disordered proteins,^79^ and has been identified as the molecular origin of the entropy convergence violation in long hydrocarbons.^80^ Following Uversky, an increase in temperature induces the partial folding of intrinsically unstructured proteins, including α-syn, rather than the unfolding typical of ordered globular proteins.^81^ This behavior can also be seen in a reduced free energy surface, computed as *F* = −*RT* ln *P*(CV_1_, CV_2_) + *C*, where *P*(CV_1_, CV_2_) is the joint probability of observing the protein’s collective variables CV_1_ and CV_2_, chosen as being *R*_g_ and SASA, and *C* is a constant chosen to be −*F*_min_ = *RT* ln *P*_max_ (CV_1_, CV_2_), and, therefore, *F ≥* 0 kJ/mol. Increasing the temperature leads to the elimination of two free energy minima at higher *R*_g_ values in the case of 2kkw, while for 2n0a, the single minimum region shifts toward lower values of both *R*_g_ and SASA. Additionally, temperature induces a destabilization of the α-helix content in 2kkw and a mild increase of the intramolecular β-sheet content, in both monomeric conformations. Whereas in 2kkw the latter spans all three domains of α-syn, in 2n0a it is more pronounced in the NACterm (residues 85–95) and the onset of the C-term. Far-UV circular dichroism (CD) spectroscopy showed an enhancement of secondary structure in α-syn and other IDPs (so-called “turned-out” behavior).^81^ These structural changes are likely driven by an increase in the hydrophobic effect.^81^ The increase of the hydration free energy of apolar groups at high temperatures induces a release of hydration water (also known as biological water) next to these groups, increasing the hydration entropy and reducing solute–water interactions.^23,78,80,82,83^

### 3.2. Protein-Peptide Recognition

We now discuss the specificity of the peptides *in trans* toward their homologous sequences in the protein, meaning how selectively the peptides interact with or recognize their corresponding sequences as opposed to other regions. The average molecular conformation of the peptides studied, in their linear (LP) and cyclic (CP) forms, is represented in Figure 2.

Figure 3 shows the protein-peptide and target-peptide (i.e., the homologous sequence *in cis*-peptide) minimum distance for the linear and cyclic peptides, for the 2kkw monomeric form of α-syn. The minimum distance is the minimum atomic distance between every atom of each residue of either the protein or the target region and the peptide. Figure S1 depicts the respective protein-peptide contact maps averaged over all trajectories. Figure 3a–c suggests that the linear peptides NACore and NACterm have a high affinity for the protein (2kkw), opposite to P1. Additionally, NACore exhibits a high specificity (Figure 3d–f), binding to amino acids in the homologous sequence in every trajectory. NACterm also interacts with the homologous sequence and nearby regions in some trajectories, whereas P1 never interacts with the homologous sequence (see also Figure S1a).

**Figure 3:**
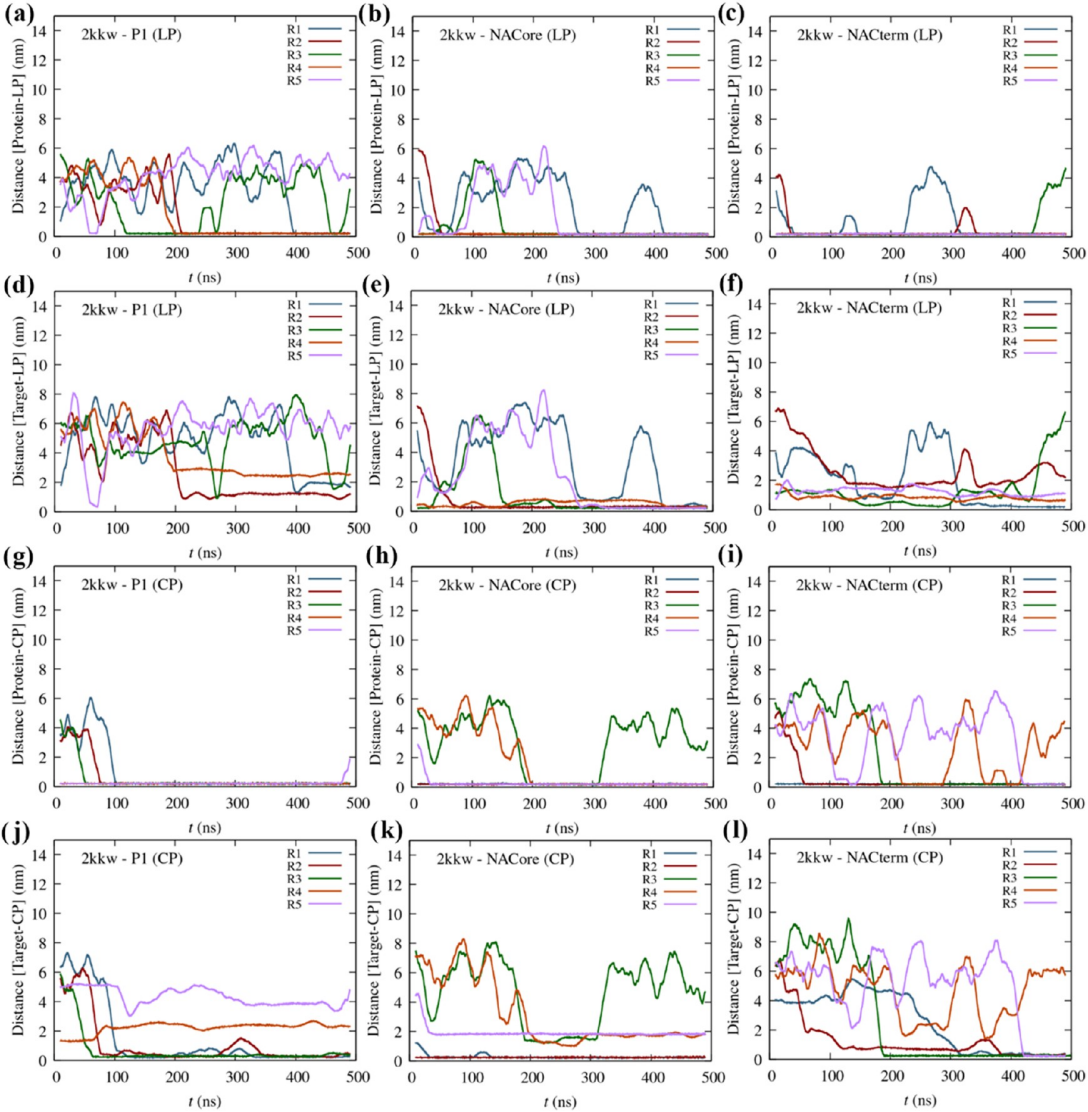
Moving averages (MA) of the minimum distance between the peptides and α-syn (2kkw) as a function of time (five replicates) for the different linear (LP) and cyclic peptides (CP): (a) P1 (LP), (b) NACore (LP), (c) NACterm (LP), (g) P1 (CP), (h) NACore (CP), and (i) NACterm (CP); MA of the minimum distance between the LPs and CPs and its homologous sequences (Target) in α-syn (2kkw) as a function of time: (d) P1 (LP), (e) NACore (LP), (f) NACterm (LP), (j) P1 (CP), (k) NACore (CP), and (l) NACterm (CP).

Concerning the cyclic peptides (Figure 3g–l), P1 exhibits a major affinity for the protein (2kkw), opposite to its linear precursor. Furthermore, the peptide interacts with its homologous region in three trajectories (Figure 3j). Because P1 has the smallest contact surface among the peptides, steric hindrance in the linear form is unlikely to explain the observed differences. Thus, the source of this seemingly opposite behavior remains unclear, although statistical limitations cannot be excluded. NACore, and especially NACterm, also exhibit specificity, with the peptides binding to their homologous sequences, respectively, in two and four trajectories. Additionally, in some trajectories, the peptides did not interact with the protein, remaining in the solvent for the duration of simulation. While the source of the observed peptide specificity cannot be fully resolved, scrambled peptide sequence controls and/or mutational analyses were not carried out due to the substantial computational cost of the simulations. We also note that, although close contact is observed between some peptides and their sequences in *cis*, this does not necessarily imply an overall interface overlap.

We now turn attention to the results for the starting conformation 2n0a (Figure 4a–l). A lower affinity for the protein is found for the linear and especially the cyclic peptides, within the simulations’ time frame.

**Figure 4:**
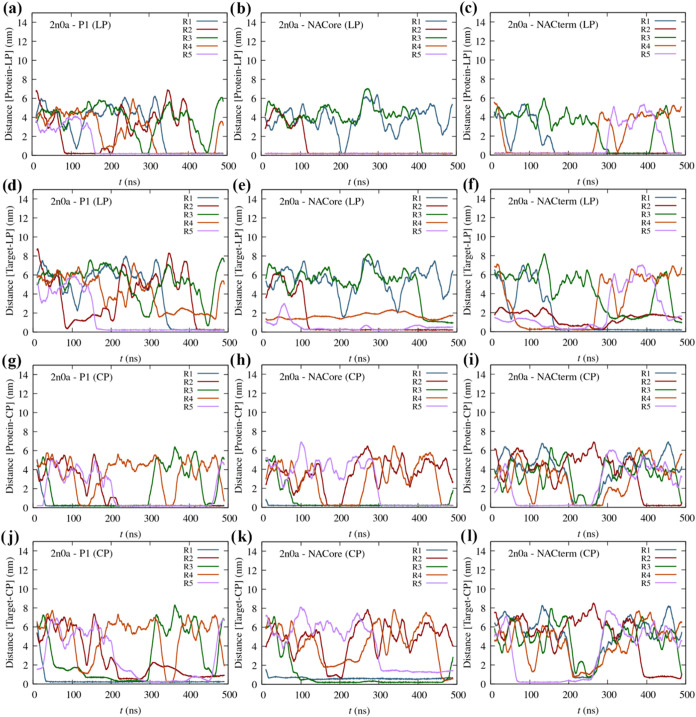
Moving averages (MA) of the minimum distance between the peptides and α-syn (2n0a) as a function of time (five trajectories) for the different linear (LP) and cyclic peptides (CP): (a) P1 (LP), (b) NACore (LP), (c) NACterm (LP), (g) P1 (CP), (h) NACore (CP), and (i) NACterm (CP); MA of the minimum distance between the LPs and CPs and its homologous sequences (target) in α-syn (2n0a) as a function of time: (d) P1 (LP), (e) NACore (LP), (f) NACterm (LP), (j) P1­(CP), (k) NACore (CP), and (l) NACterm (CP).

A lower specificity toward their homologous sequences can also be seen, relative to the 2kkw conformation. The reason is likely that the more hydrophilic C-term is more exposed whereas the homologous sequences in the NAC and the N-term regions are involved in intramolecular interactions (see Figure 1g). Thus, possibly longer simulations would be required to observe more protein-peptide binding events. Nevertheless, in most trajectories where the peptides interact with the protein it can be seen that these interactions (i.e., minimum distances) occurred with the homologous sequence or a nearby region, especially for the linear peptides and cyclic P1 (see also Figure S2). Interestingly, a lower number of protein-peptide interaction events is also observed between linear P1 and 2n0a, similar to the 2kkw monomer. A possible reason for this behavior could be the smaller size of P1 (7 amino acids) relative to NACore and NACterm (11 amino acids). However, again, insufficient sampling cannot be ruled out.

In spite of sampling and force field limitations for IDPs these results suggest that MD simulations can capture the protein-peptide specificity assumed in the design of synthetic peptide aggregation inhibitors, supporting the view that aggregation is inhibited through a direct PPI blocking mechanism. We stress that, with the exception of linear P1, which is not expected to prevent aggregation,^39^ the influence of the peptides on aggregation is not known, and is beyond the scope of this study.

### 3.3. Peptide Influence on α-Syn’s Structure

While it remains unclear how function derives from disordered states in IDPs, more limited conformations are expected when bound to their biological targets.^84^ Thus, it is important to understand whether protein-peptide binding also promotes more limited conformations in addition to block segments involved in the formation of cross β-sheets. Conversely, such binding may instead favor aggregation by disrupting stabilizing intramolecular interactions.^35,39^ We stress, nevertheless, that the influence of any structural transformation of the monomer on the protein’s function remains elusive.

We assessed the influence of the peptides on the conformational space of the protein by analyzing the time windows when the peptides were bound to the protein homologous sequences. Table S1 shows the selected trajectories and time segments for the linear and cyclic peptides for the 2kkw starting conformation, and Figure 5 shows the respective contact maps.

**Figure 5:**
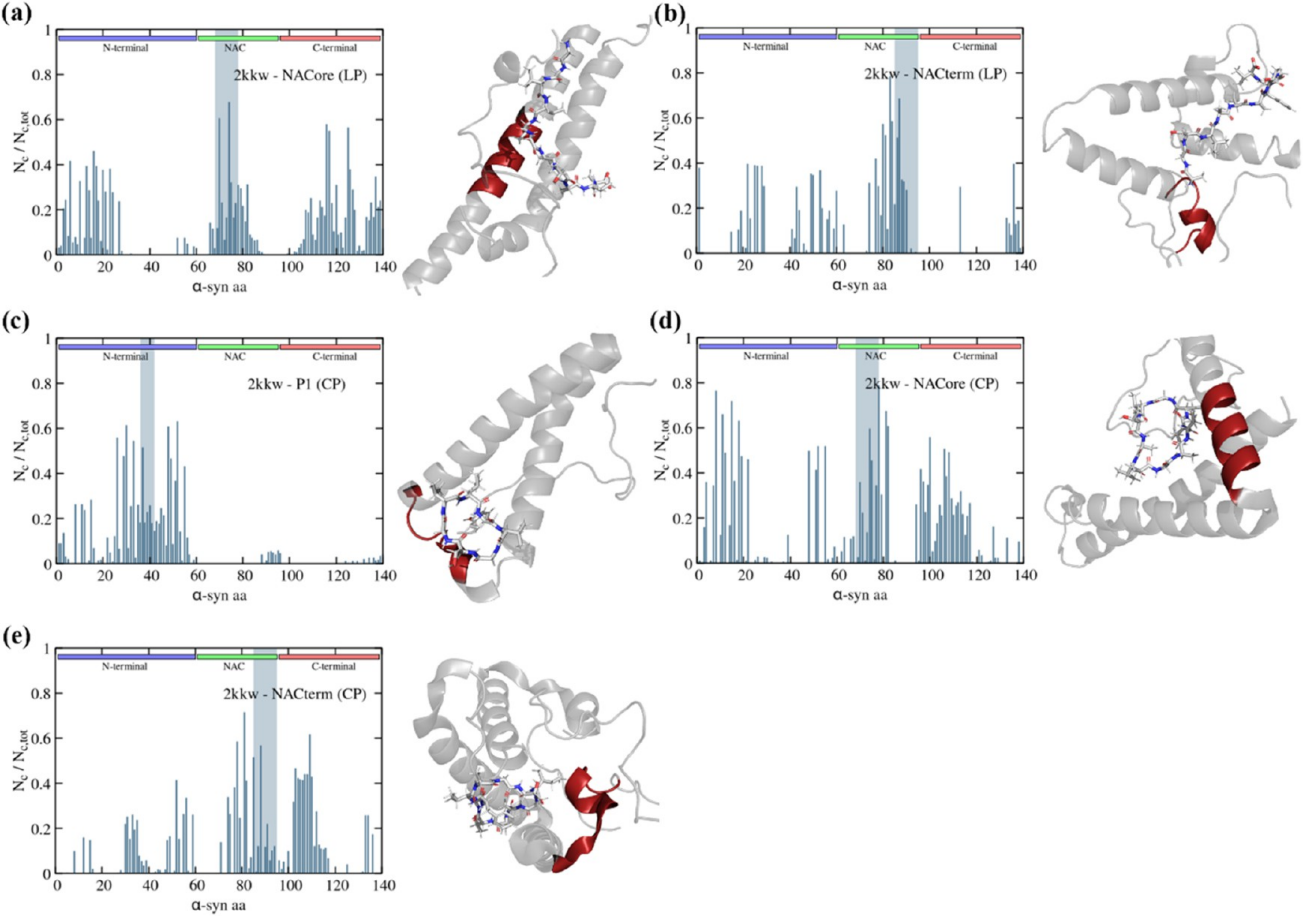
Protein-peptide contact maps and respective representation of α-syn (2kkw): (a) NACore (LP), (b) NACterm (LP), (c) P1 (CP), (d) NACore (CP), and (e) NACterm (CP); the homologous sequences are represented by a gray vertical stripe. Linear P1 is omitted since it did not bind to the homologous sequence. A MD snapshot of the respective peptides bond to the protein is depicted; the homologous in cis sequences are depicted in red.

The linear NACore showed the highest specificity among the peptides studied (see Figure 3e). Figure 5a shows, however, that in spite of the minimum protein-peptide distance, in the selected time-windows, being observed for the homologous region, the peptide interacts (i.e., distance protein-peptide < 3.5 Å) concomitantly with several other regions. This is explained by the protein conformation, also accounting for the apparent large number of contacts depicted in Figures S1 and S2 where the averages include the time the peptides were in contact with any region of the protein as well as in the solvent. A similar picture can be observed for NACterm (LP), with the peptide contacting with all three regions of α-syn. For the cyclic peptides, P1 shows a high specificity toward its homologous region without significantly interacting with the NAC (amino acids 61–95) and the C-term (amino acids 96–140). For cyclic NACore and NACterm the situation is more similar to that observed for their linear precursors, with pronounced interactions with other amino acids, especially in the C-term domain. We stress that the latter has a higher mobility, relative to the N-term and the NAC, in the 2kkw conformation, allowing it to interact with the peptide even when this is lodged in the homologous region.

A similar behavior is observed for 2n0a (see Figure 6); Table S2 shows the respective selected trajectories and time segments. The smaller number of interactions with the protein (i.e., less scattered) is observed for cyclic NACore and NACterm. However, this is probably due to the fact that binding to the homologous sequence was observed for a single trajectory (see Figures 4k,l).

**Figure 6:**
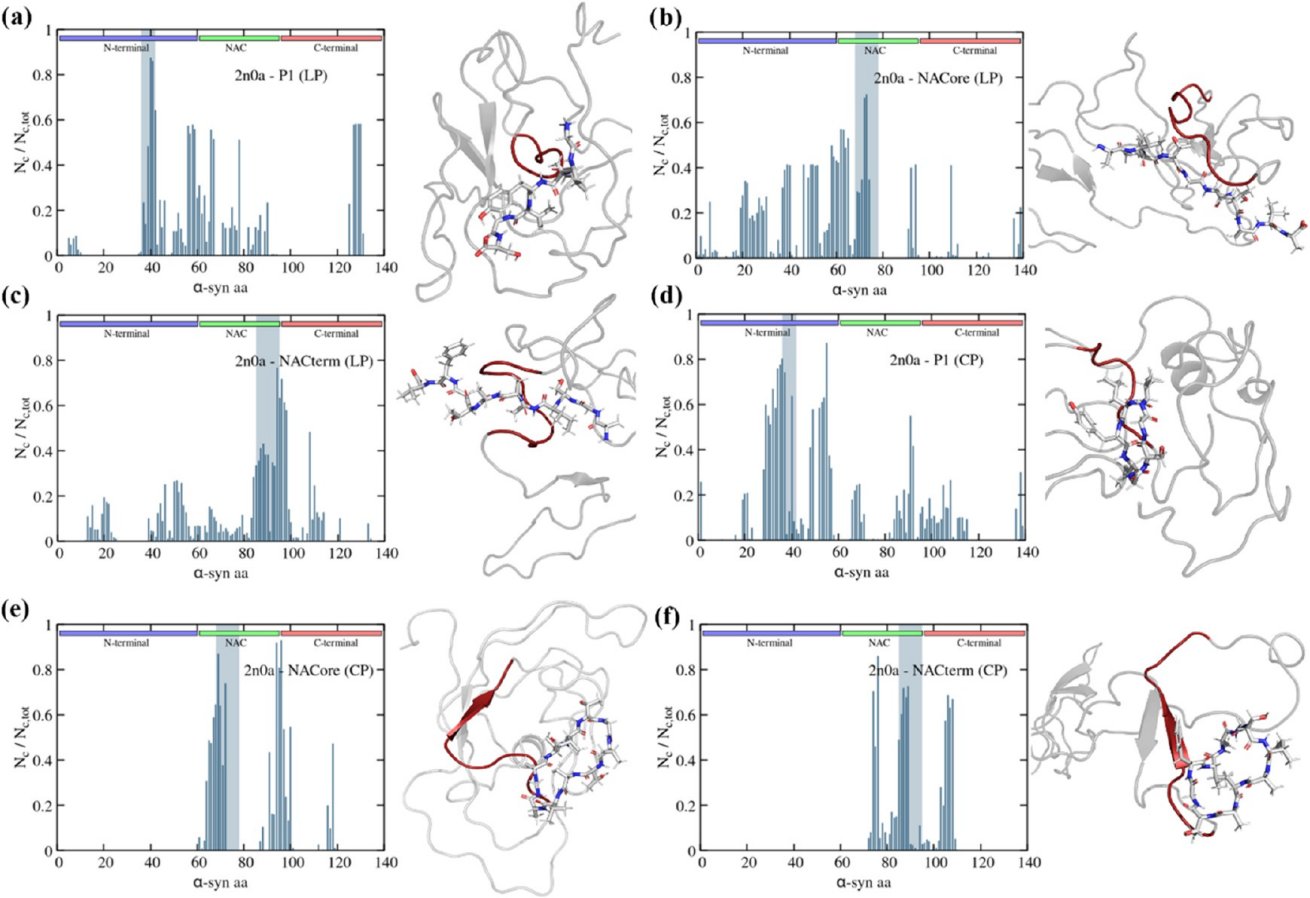
Protein-peptide contact maps and respective representation of α-syn (2n0a): (a) P1 (LP), (b) NACore (LP), (c) NACterm (LP), (d) P1 (CP), (e) NACore (CP), and (f) NACterm (CP); the homologous sequences are represented by a gray vertical stripe. Cyclic NACore is omitted since it did not bind to the homologous sequence. A MD snapshot of the respective peptides bond to the protein is depicted; the homologous in cis sequences are depicted in red.

We now analyze the influence of the contacts depicted in Figures 5 and 6 on the structure of α-syn. Figure 7 shows *R*_g_ and SASA distributions for the 2kkw (Figure 7a–f) and 2n0a (Figure 7g–l) conformations, when the peptides are in close contact with the homologous sequences in the protein. Similar plots, averaged over the last 250 ns of all the trajectories, independent of the peptides’ position, are reported in Figure S3. Unlike the effect of temperature, the peptides do not cause a shift of *R*_*g*_ and SASA toward lower values. Thus, instead, for 2kkw a small shift to larger values (i.e., more extended conformations) is observed for some peptides (P1 (CP), NACore (LP)). Additionally, reduction of some intermediate *R*_*g*_ values is observed for cyclic NACore and NACterm without, however, a major impact in the SASA. This behavior results in the appearance of a region for α-syn with NACterm (CP) where the joint probability of observing some *R*_*g*_ and SASA values is almost zero, resulting in a marked free energy barrier between minima (see Figure S4).

**Figure 7:**
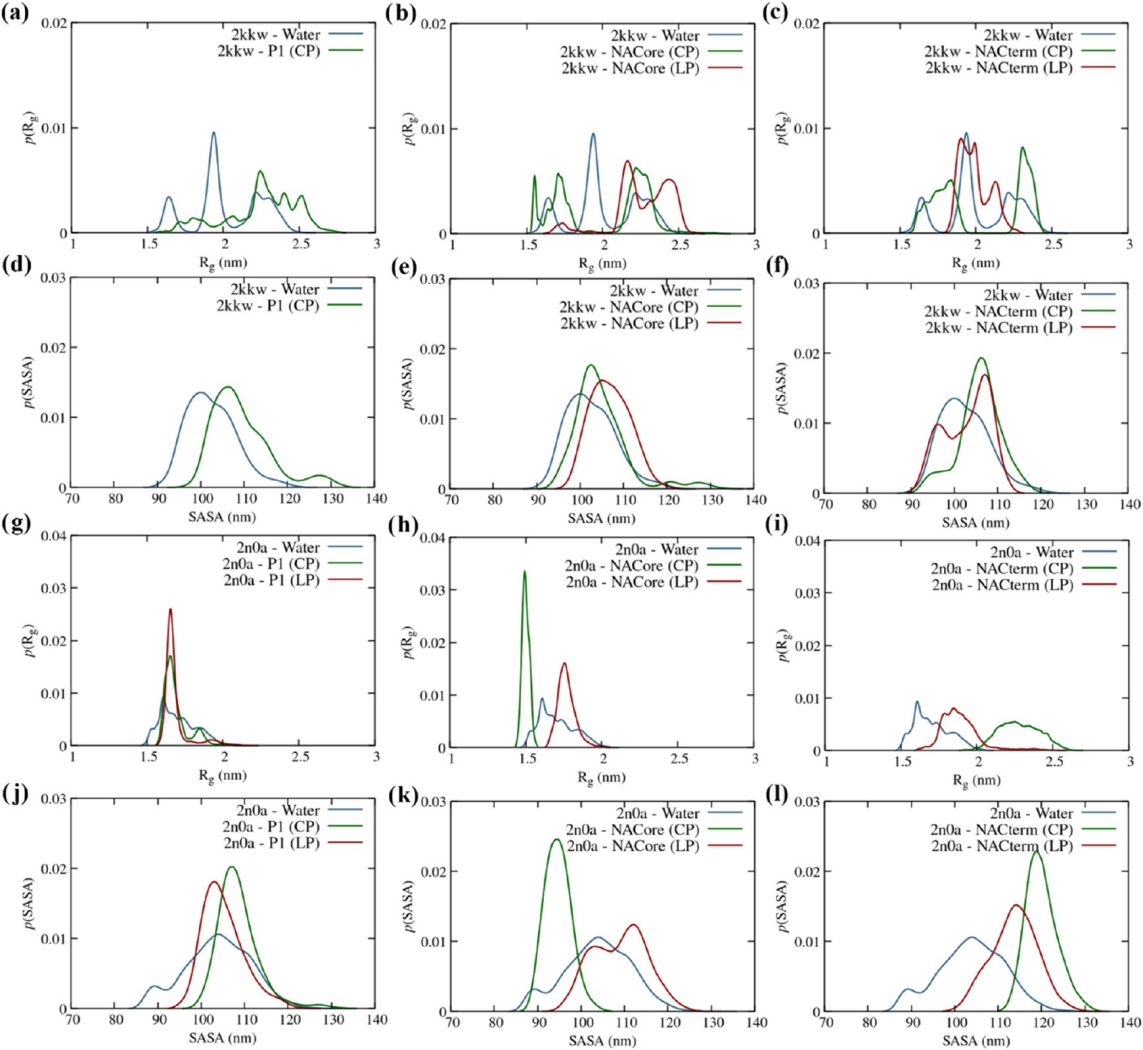
Radius of gyration distribution functions for 2kkw with (a) P1, (b) NACore, and (c) NACterm for the selected trajectories. SASA distribution functions for 2kkw with (d) P1, (e) NACore, and (f) NACterm for the selected trajectories. Radius of gyration distribution functions for 2n0a with (g) P1, (h) NACore, and (i) NACterm for the selected trajectories. SASA distribution functions for 2n0a with (j) P1, (k) NACore, and (l) NACterm for the selected trajectories.

A similar behavior can be seen for the 2n0a starting conformation concerning *R*_g_ and the SASA with the exception of NACore (CP) for which a shift to lower values and pronounced narrowing is observed (Figure 7h). The shift to lower values of *R*_g_ for NACore (CP) (based on a single trajectory) is seemingly associated with an increase in the α-helix in the N-term and NAC domains (Figure 9b). The most prominent shift to more extended structures is observed for linear and cyclic NACterm, although the latter is also based on a single trajectory (Figure 7l). Averaging over all trajectories, independent of the peptide location, resolves these shifts for NACore (CP) and NACterm (CP) (see Figure S3h,i) but not for linear NACterm (Figure S3i).

For P1 (LP) a narrowing of the *R*_g_ and SASA distributions can be seen, suggesting a reduction of the conformational space of α-syn. Moreover, this narrowing persists when the *R*_g_ and the SASA are averaged over all trajectories (Figure S3g).

Figures 8 and 9 show the α-helix and β-sheet distributions for the 2kkw and 2n0a starting conformations, respectively, when the peptides are in close contact with the homologous sequences in the protein. Similar plots averaged over the last 250 ns of all the trajectories, independent of the peptides’ position, are reported in Figures S5 and S6. An increase in the α-helix content is observed between residues 72 and 92 (Figure 8a–e), supporting the idea of stabilization of the membrane-bond structure (2kkw) (light green areas). For NACterm (LP) this enhancement is confined to the residues 74–84. However, some α-helix destabilization is also observed in the N-term for some peptides and a significant reduction around amino acid 67, particularly prominent for the linear NACterm (Figure 8e). The latter shows α-helix “nodes” at residues 67–68 and residues 85–87. A minor increase in β-sheet, in turn, is only observed for residues in the C-term, consistent with the fact that the protein preserves its α-helices, not forming significant intramolecular or intermolecular β-sheets (Figure S7 displays the distribution of intermolecular β-sheets).

**Figure 8:**
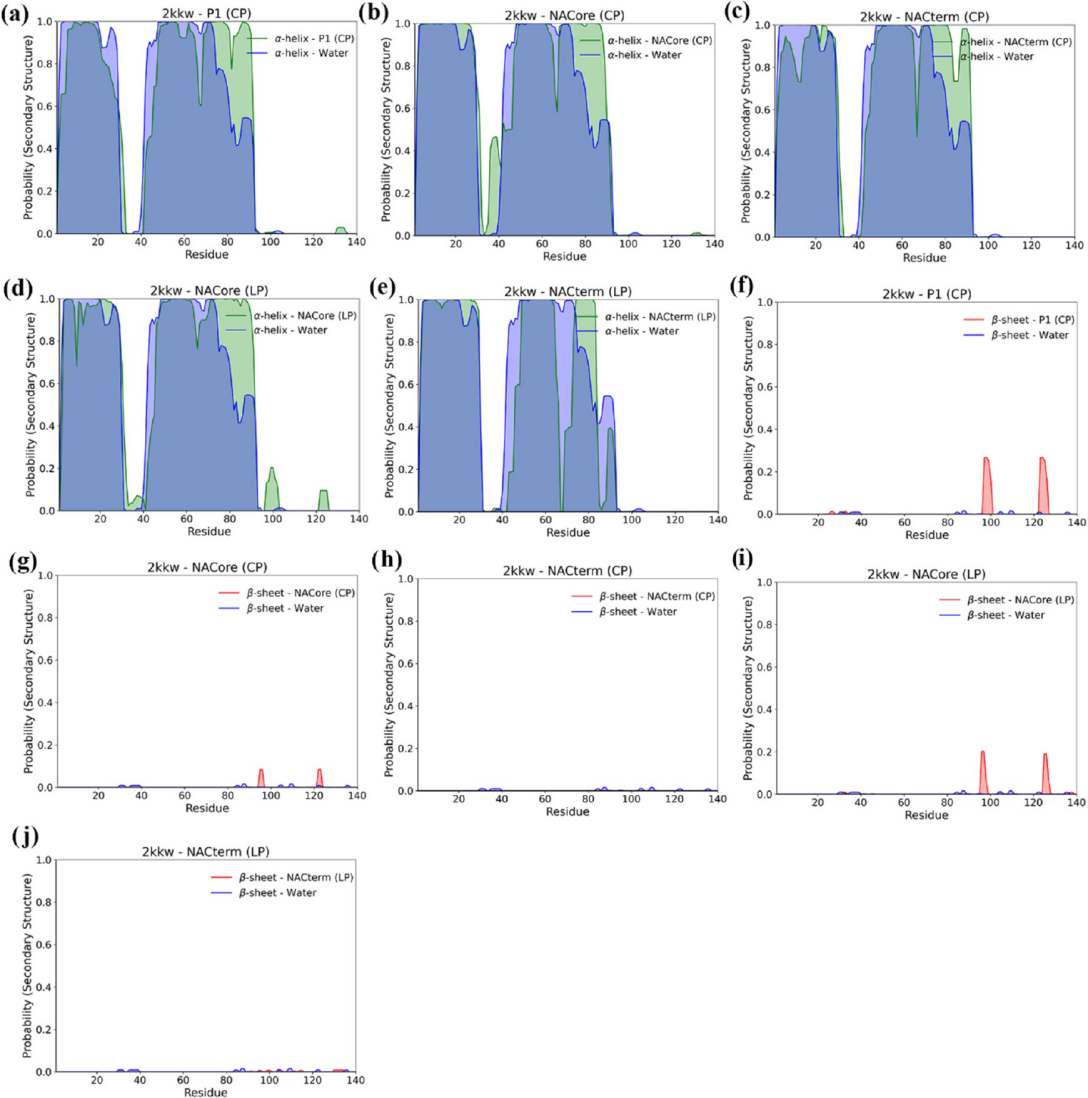
α-helix (a–e) and β-sheet (f–j) distributions per residue for the selected trajectories, for the linear and cyclic peptides and the 2kkw starting conformation. The dark blue shading corresponds to intersection regions of α-helix observed both with and without the peptide. The visible green regions represent the α-helix regions exclusively observed in the presence of the peptide.

**Figure 9:**
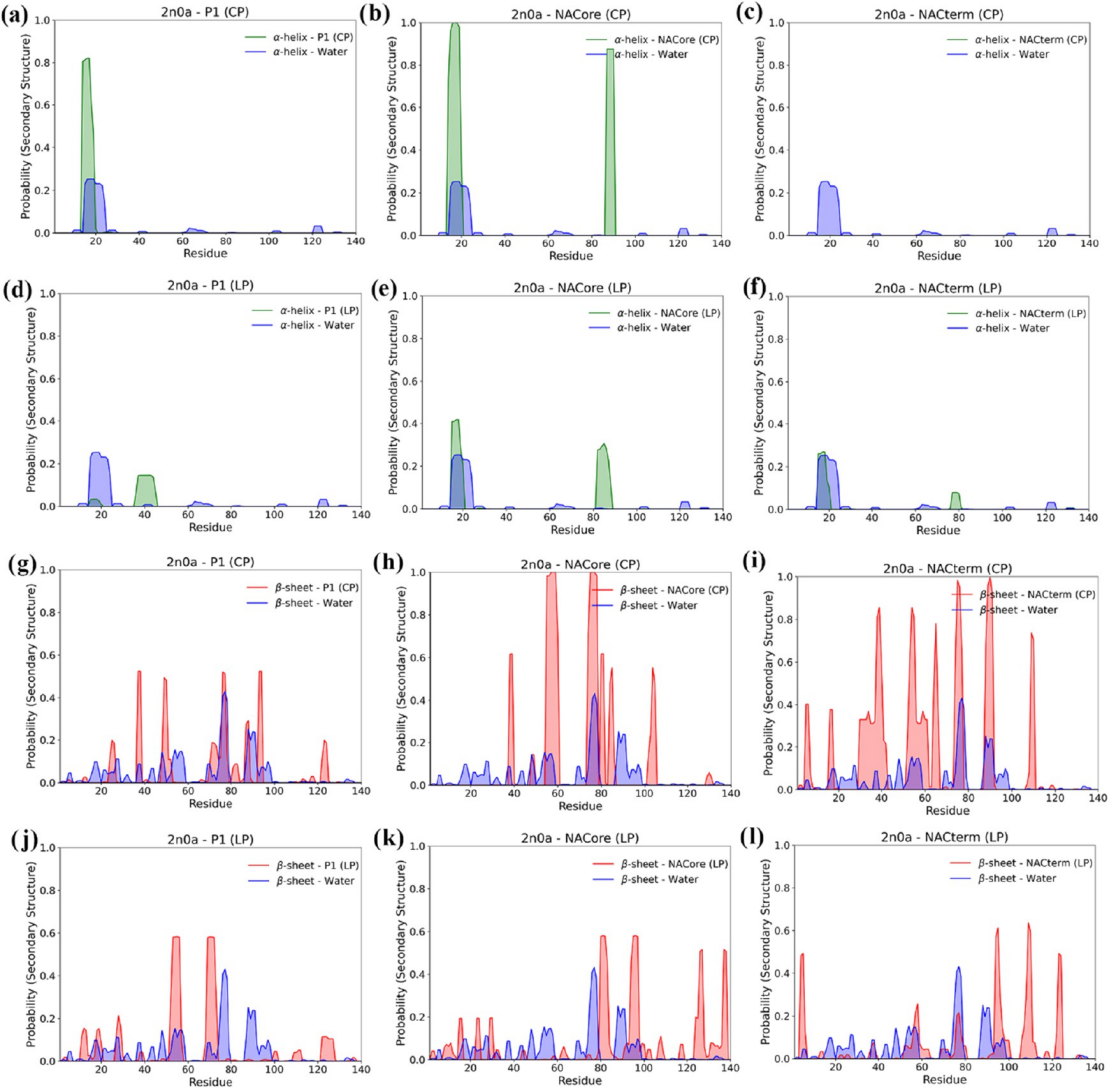
α-helix (a–f) and β-sheet (g–l) distributions per residue for the selected trajectories, for the linear and cyclic peptides and the 2n0a starting conformation. The dark blue shading corresponds to intersection regions of α-helix observed both with and without the peptide. The visible green regions represent the α-helix regions exclusively observed in the presence of the peptide.

As expected, no significant α-helix forms, associated with protein-peptide interactions. However, unlike for 2kkw an increase of the β-sheet is observed for 2n0a. This is more marked for NACterm (CP) and NACore (CP) displayed in Figure 9h,i. However, the observed β-sheet is predominantly intramolecular (see Figure S7), indicating that the peptides do not form extensive intermolecular β-sheets with the protein. Whether such an increase of the intramolecular β-sheet is an indication of a greater aggregation tendency remains elusive.

The observation that some peptides increase α-syn’s solvent exposure upon binding to their homologous region suggests a reduction in the hydrophobic effect, potentially reducing α-syn’s aggregation propensity. However, this might also result from the disruption of α-syn’s intramolecular interactions which may play a protective role against aggregation.^35,39^ Thus, assessing α-syn’s aggregation propensity based on the structural transformations of the monomer alone can be misleading. We note, however, that studying α-syn aggregation (or its inhibition) through MD, remains challenging, owing to system size limitations. Thus, MD simulations can only probe α-syn (and other proteins) at concentrations near or above their saturation concentration. As a result, aggregation may still occur even in the presence of unrealistically high concentrations of inhibitors.

To gain additional insight into intramolecular interactions we analyzed the distances across α-syn’s C_α_ over the same trajectory time-windows (see Table S1 and S2).

Figures 10 and 11 show the respective distance heat maps for 2kkw and 2n0a. The most prominent result for 2kkw is the generalized reduction of the intramolecular distances observed when linear and especially cyclic NACterm are bound to its homologous sequence in α-syn. This is difficult to conclude from the *R*_g_ and SASA distributions (see Figure 7c,f). A less pronounced effect is observed for NACore (CP). The other most notable result is the P1 (CP)-induced increase in the intramolecular distances between the 60–120 region and the C-term (Figure 10d), opposite to the effect of NACore (CP) and NACterm (CP) (Figure 10e,f). A similar, although less pronounced, intramolecular distances increase can be seen for NACterm (LP).

**Figure 10:**
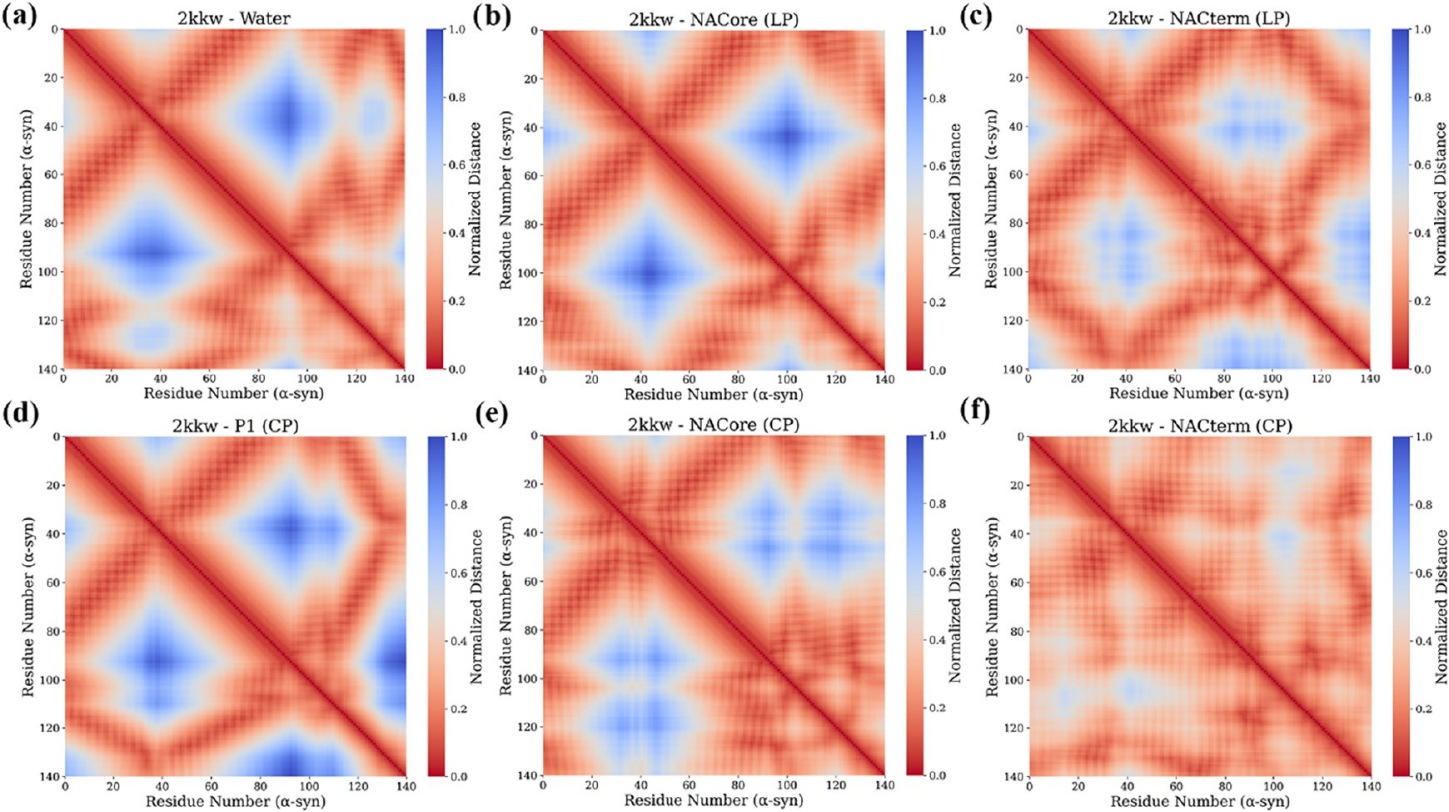
α-syn Cα distance maps for the 2kkw systems: (a) aqueous solution, (b) NACore (LP), (c) NACterm (LP), (d) P1 (CP), (e) NACore (CP) and (f) NACterm (CP). The distances were normalized by the largest average distance between a pair of residues. The latter was found for the P1 (CP) system (∼8.3 nm).

**Figure 11:**
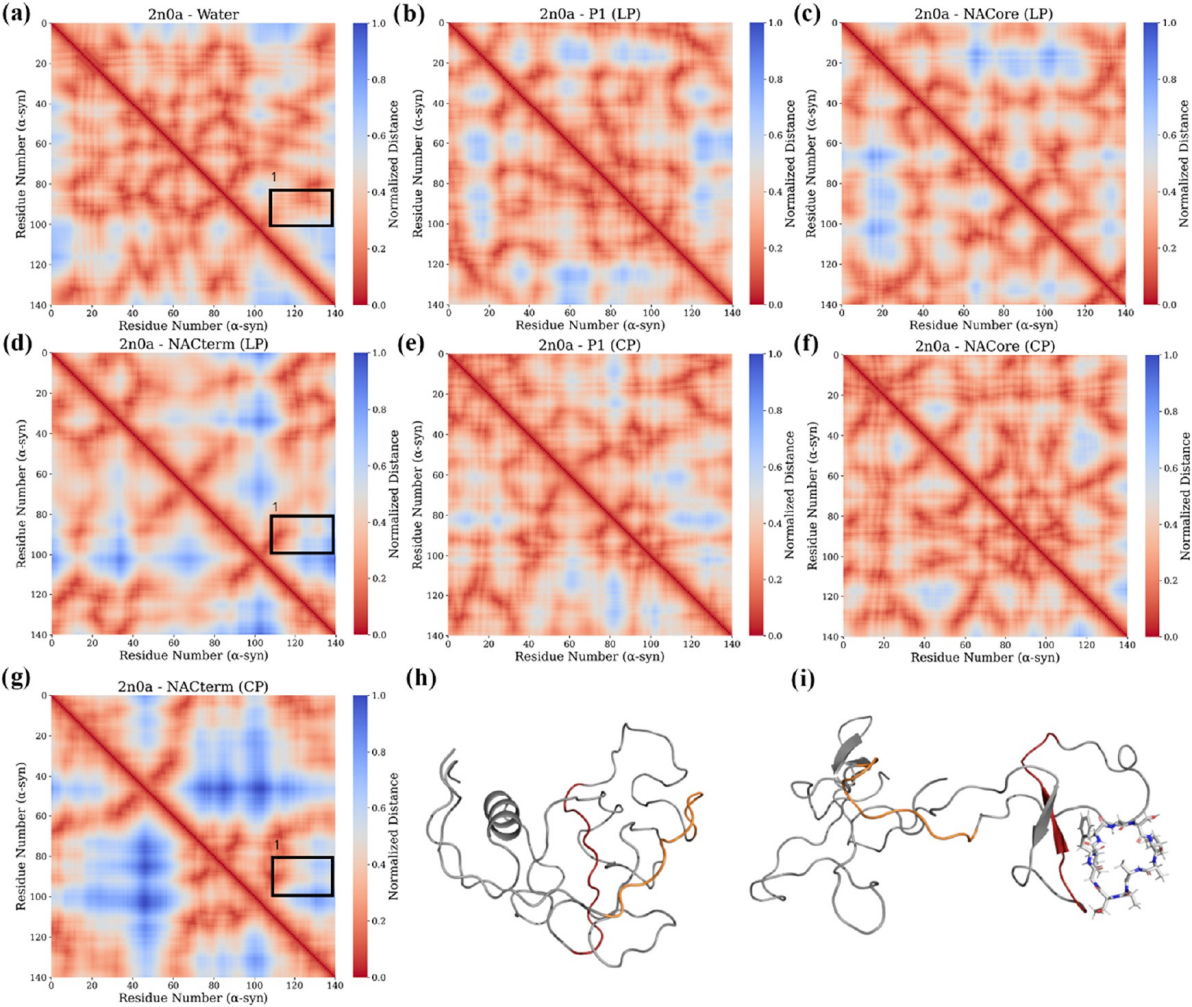
α-syn Cα distance maps for the 2n0a systems: (a) aqueous solution, (b) P1 (LP), (c) NACore (LP), (d) NACterm (LP), (e) P1 (CP) and (f) NACore (CP), and (g) NACterm (CP). The distances were normalized by the largest average distance between a pair of residues. The latter was found for the NACterm (CP) system (∼6.6 nm). (h) and (i) represent α-syn conformations in the absence of any peptide and when bound to NACterm (CP), respectively, illustrating the increased distances between the NACterm in cis (in red) and the C-term (in orange), corresponding to the rectangle in (a), (d), and (g).

For 2n0a an opposite picture can be seen, with NACterm (CP) and to a less extent NACterm (LP) inducing longer intramolecular distances between residues in the N-term and residues in the NAC and C-term. This is consistent with the increase of the *R*_g_ and SASA observed in Figure 7i,l, indicating that the same peptide may have different effects over different conformations of α-syn, namely, when in solution and when membrane-bound. Furthermore, these results suggest that cyclic NACterm might both eliminate protective intramolecular interactions - mainly the contacts between NACterm *in cis* and C-term residues such as Met^116^, Val^118^, Tyr^125^, and Met^127^ (Figure 11a,d,g represented by a black rectangle^35^) - favoring aggregation, and stabilize the monomer as reflected in a larger SASA, opposing aggregation. Nevertheless, again, a single trajectory was found where NACterm (CP) is bound to the homologous region (see Table S2).

Although binding preferentially to the N-term and the NAC, linear P1, modified for enhanced solubility (Ac-KTKE-_36_GVLYVGS_42_–KTKE-NH2), was experimentally shown to destabilize protective intramolecular interactions between the C-term and the α-syn P1 *in cis*.^39^ Here, linear P1 *in trans* (zwitterionic) exhibited affinity for the C-term region (around residues 125–131) in the 2n0a starting conformation simulations (see Figure 6a), in addition to the pre-NAC and NAC regions. Thus, an increase of the distances between the pre-NAC/NAC and the C-term is observed for linear P1 and the 2n0a conformation (Figure 11a,b). This supports the view that the protective role of intramolecular interactions between the N-term and C-term domains might be compromised by P1 *in trans*.^39,51^

While our results provide evidence of the specificity of cyclic peptides and their linear precursors toward α-syn in both membrane-bound and disordered forms, as well as their impact on the protein’s structure, they do not allow us to draw conclusions about the peptides’ potential modulator and inhibitory activity. The study of protein aggregation of IDPs through MD poses several challenges because of the impossibility of distinguishing between neurotoxic and non-neurotoxic oligomers in addition to system size and sampling limitations and was not pursued in this study.

## 4 Conclusions

The aggregation of α-syn is implicated in various synucleinopathies. Experimental mutagenesis studies have identified several key aggregation-prone regions, particularly in the N-terminal and NAC domains. The development of drugs targeting these regions represents, therefore, a potential therapeutic strategy. However, blocking these regions may also play the opposite effect, eliminating protective intramolecular interactions. Molecular dynamics simulations can provide atomic-level insight into the interactions between potential drugs and these domains, including their specificity toward specific targets and their influence on the protein’s conformational space. Here, we studied the specificity of several cyclic peptides and their linear precursors toward their homologous sequences of α-syn. The results show that both linear and cyclic peptides exhibit specificity toward a membrane-bond and disordered conformations of α-syn. A stabilization of specific α-helical regions of the membrane-bound conformation is observed which may contribute to the inhibition of aggregation. However, peptide binding can also weaken specific intramolecular interactions in the disordered form of α-syn, potentially promoting aggregation-prone conformations. Therefore, peptide design, whether de novo or derived from amyloidogenic sequences, should balance aggregation inhibition with the preservation of protective intramolecular contacts. This balance may be achieved by avoiding sequences that stabilize the native protein ensemble and/or by preferentially interacting with specific conformational states. We stress, nevertheless, that a complete characterization of such conformational states remains elusive.

In spite of the above limitations our study reveals that linear and cyclic peptides may be used as protein recognition elements coupled with amyloid aggregation modulators or inhibitors and that these can be studied through molecular dynamics simulations. The peptides investigated here can be further evaluated using a number of experimental techniques, including Thioflavin T fluorescence assays to determine their influence on the aggregation kinetics of α-syn. These peptides may serve as models for the formation of nontoxic oligomeric species of α-syn, potentially offering insight into protective aggregation pathways.

## Supplementary Material



## Notes

Tables of peptide–protein interaction time-windows; protein–peptide contact maps, radius of gyration (*R*_g_) and solvent-accessible surface area (SASA) distributions, and per-residue α-helix and β-sheet distributions, including intra- and intermolecular β-sheet analyses are displayed in the SI (PDF)
