# Supplementary material for: Protein Recognition of Linear and Cyclic Peptides of Homologous Sequences Implicated in the Aggregation of α‑Synuclein
Source: J Phys Chem B. 2025 Oct 13;129(42):11015–30. doi: 10.1021/acs.jpcb.5c05501 (PMC12818748; doi:10.1021/acs.jpcb.5c05501)
Supplement: Supplementary file 1 [file jp5c05501_si_001.pdf]

Protein Recognition of Linear and Cyclic Peptides of Homologous Sequences Implicated in the Aggregation of α-Synuclein

Supporting Information

Gabriel F. Martins[a], Cristiano Rocha[a], Nuno Galamba[a*]

[a] BioISI - Biosystems and Integrative Sciences Institute, Faculty of Sciences of the University of Lisbon, C8, Campo Grande, 1749-016 Lisbon, Portugal.

*Corresponding author: njgalamba@fc.ul.pt

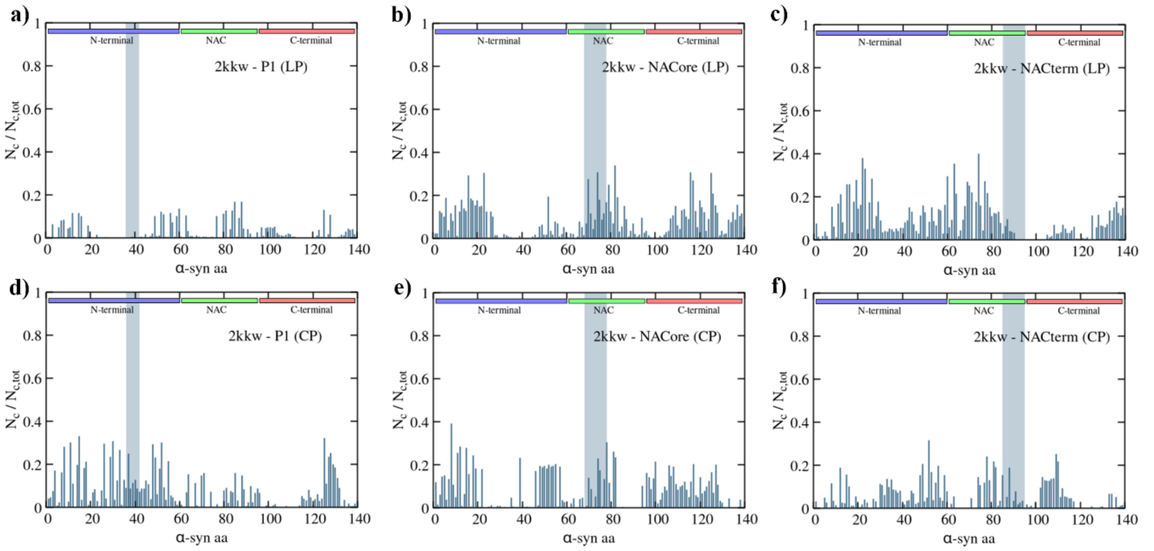

Figure S1 – Protein-peptide contact maps for α-syn (2kkw): **(a)** P1 (LP), **(b)** NACore (LP), **(c)** NACterm (LP), **(d)** P1 (CP), **(e)** NACore (CP), and **(f)** NACterm (CP). A 3.5 Å cut-off distance was used to define the protein-peptide contacts. The respective homologous sequences *in cis* are represented by a gray vertical bar.

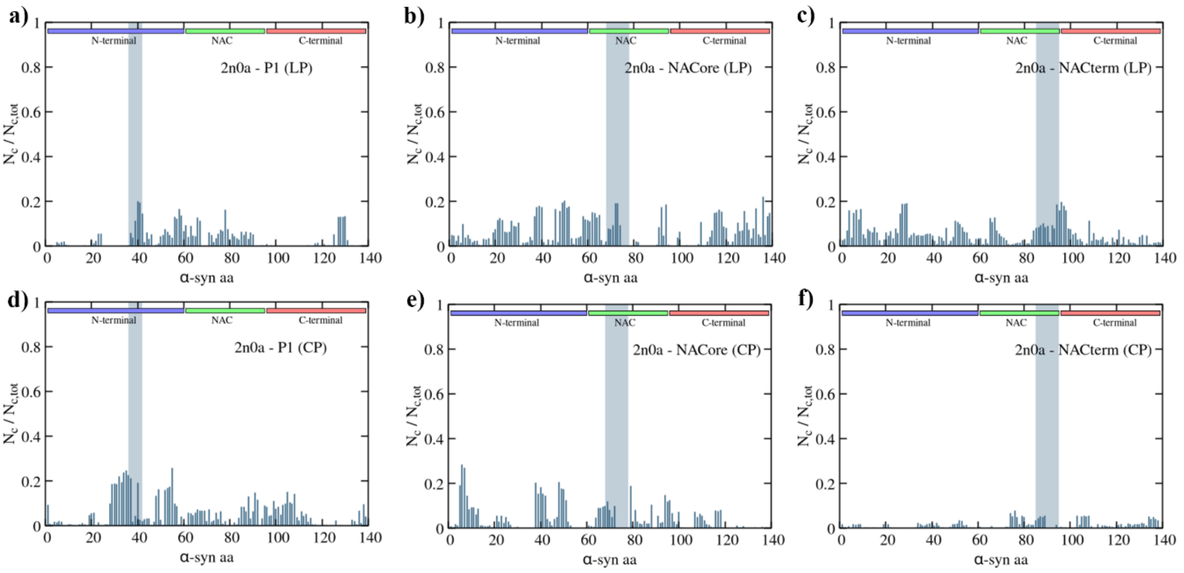

Figure S2 – Protein-peptide contact maps for α-syn (2n0a): **(a)** P1-LP, **(b)** NACore (LP), **(c)** NACterm (LP), **(d)** P1 (CP), **(e)** NACore (CP), and **(f)** NACterm (CP). A cut-off distance of 3.5 Å was used to define the protein-peptide contacts. The respective homologous sequences *in cis* are represented by a gray vertical bar.

Table S1 – Trajectories and time-windows where the peptides interacted with their respective homologous sequence on α-syn (2kkw).

System	Trajectory (Time Window)
2kkw + P1(LP)	n/a
2kkw + P1(CP)	R1 (120 – 500 ns)<br>R2 (80 – 500 ns)<br>R3 (90 – 500 ns)
2kkw + NACore(LP)	R1 (420 – 500 ns)<br>R2 (80 – 500 ns)<br>R3 (190 – 500 ns)<br>R4 (420 – 500 ns)<br>R5 (280 – 500 ns)
2kkw + NACore(CP)	R1 (0 – 500 ns)<br>R2 (0 – 500 ns)
2kkw + NACterm(LP)	R1 (320 – 500 ns)<br>R2 (180 – 300 ns)
2kkw + NACterm(CP)	R1 (320 – 500 ns)<br>R2 (390 – 500 ns)<br>R3 (190 – 500 ns)<br>R4 (420 – 500 ns)

Table S2 – Trajectories and time-windows where the peptides interacted with their respective homologous sequence on α-syn (2n0a).

System	Trajectory (Time Window)
2n0a + P1(LP)	R1 (350 – 500 ns)<br>R2 (420 – 500 ns)<br>R3 (180 – 500 ns)
2n0a + P1(CP)	R1 (50 – 500 ns)<br>R3 (220 – 290 ns)<br>R5 (290 – 450 ns)
2n0a + NACore(LP)	R1 (120 – 500 ns)<br>R2 (110 – 380 ns)
2n0a + NACore(CP)	R3 (150 – 470 ns)
2n0a + NACterm(LP)	R1 (180 – 500 ns)<br>R4 (100 – 250 ns)<br>R5 (190 – 250ns)
2n0a + NACterm(CP)	R1 (80 – 250 ns)

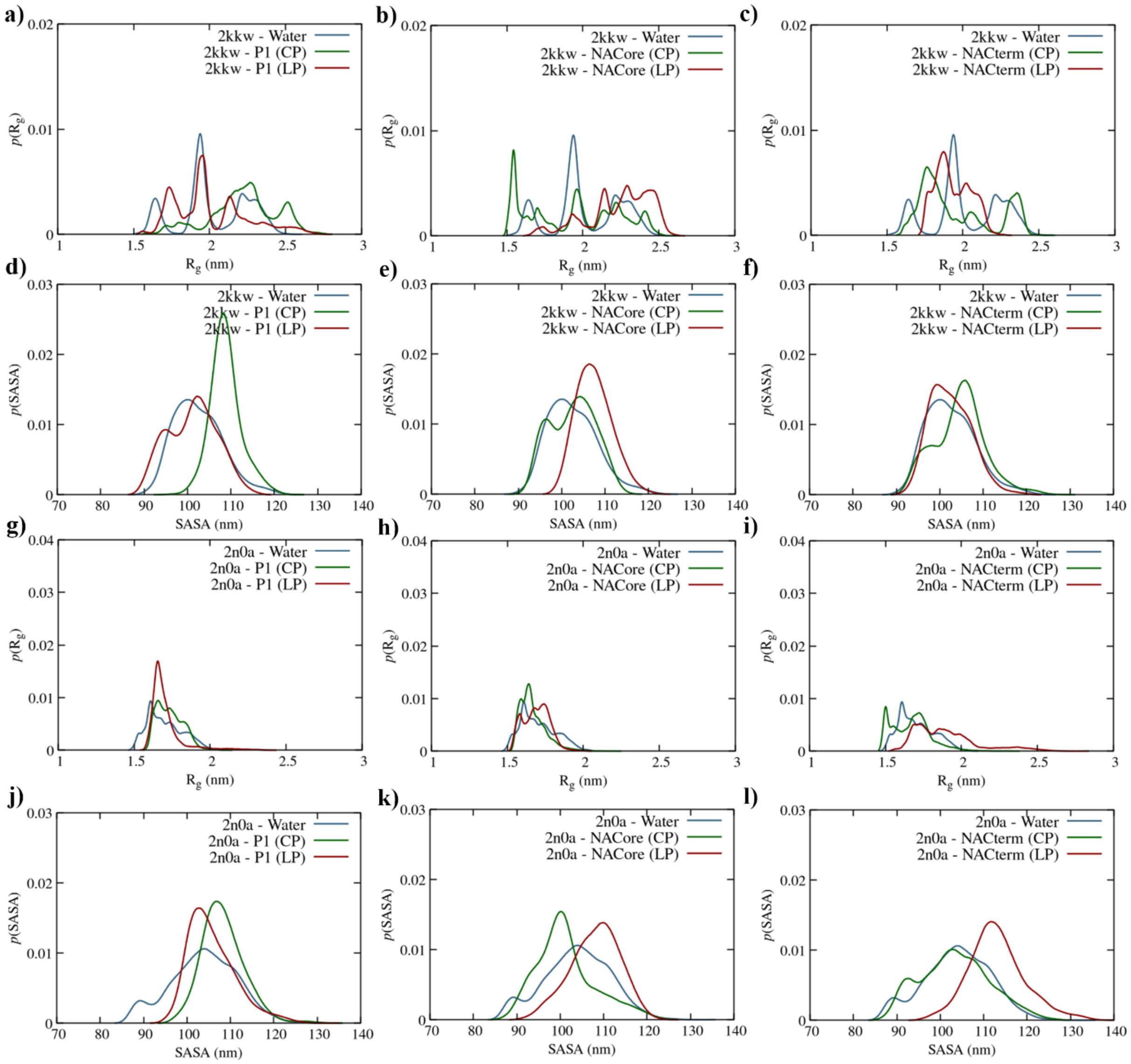

Figure S3 – Radius of gyration distribution functions for 2kkw with **(a)** P1, **(b)** NACore, and **(c)** NACterm averaged over the last 250 ns of all trajectories. SASA distribution functions for 2kkw with **(d)** P1, **(e)** NACore, and **(f)** NACterm averaged over the last 250 ns of all trajectories. Radius of gyration distribution functions for 2n0a with **(g)** P1, **(h)** NACore, and **(i)** NACterm averaged over the last 250 ns of all trajectories. SASA distribution functions for 2n0a with **(j)** P1, **(k)** NACore, and **(l)** NACterm averaged over the last 250 ns of all trajectories.

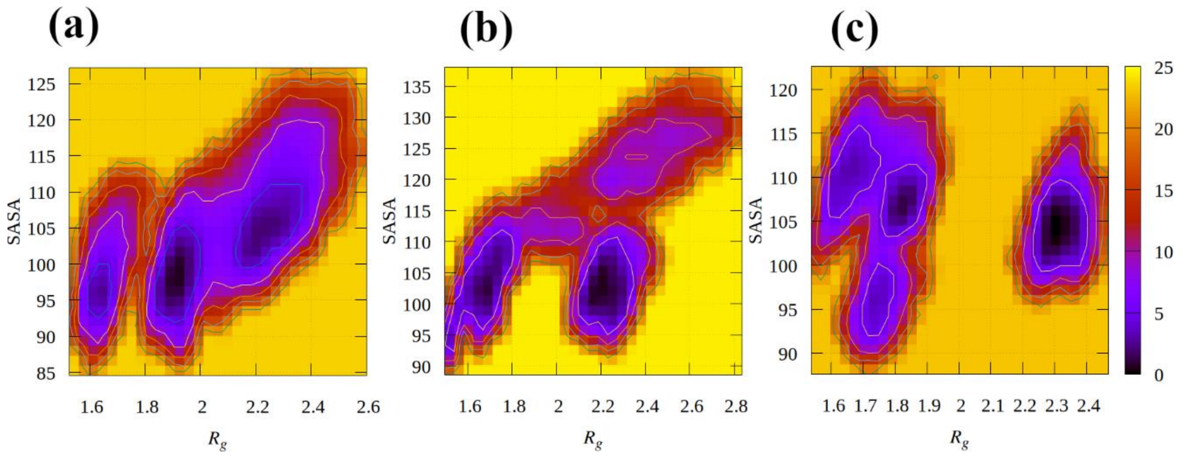

Figure S4 – Reduced free energy surface for α-syn (2kkw) in the absence of peptides **(a)** and when bound to cyclic **(b)** NACore and **(c)** NACterm.

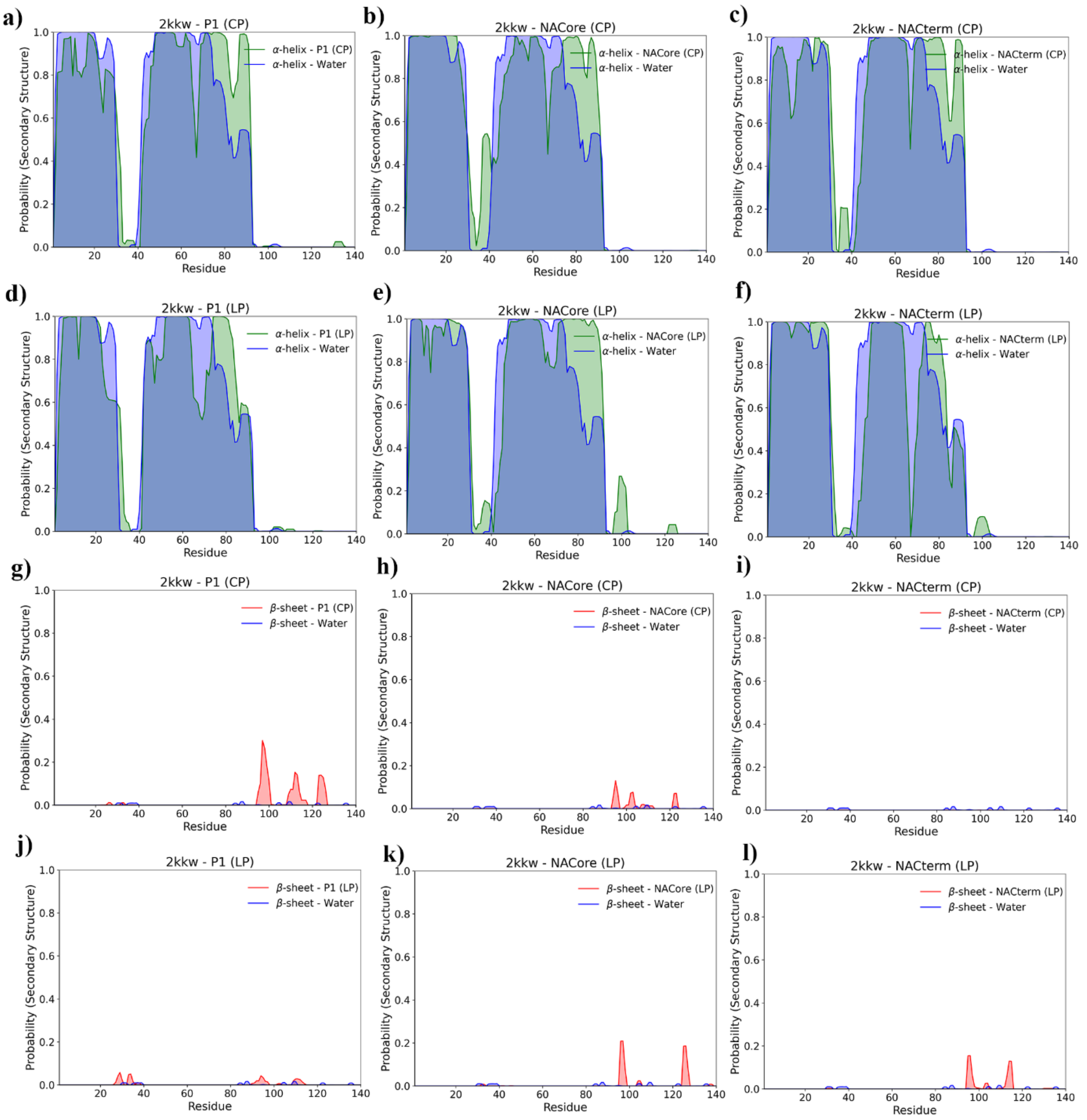

Figure S5 – α-helix **(a-f)** and β-sheet **(g-l)** distributions per residue, averaged over the last 250 ns of all trajectories, for the linear and cyclic peptides and 2kkw.

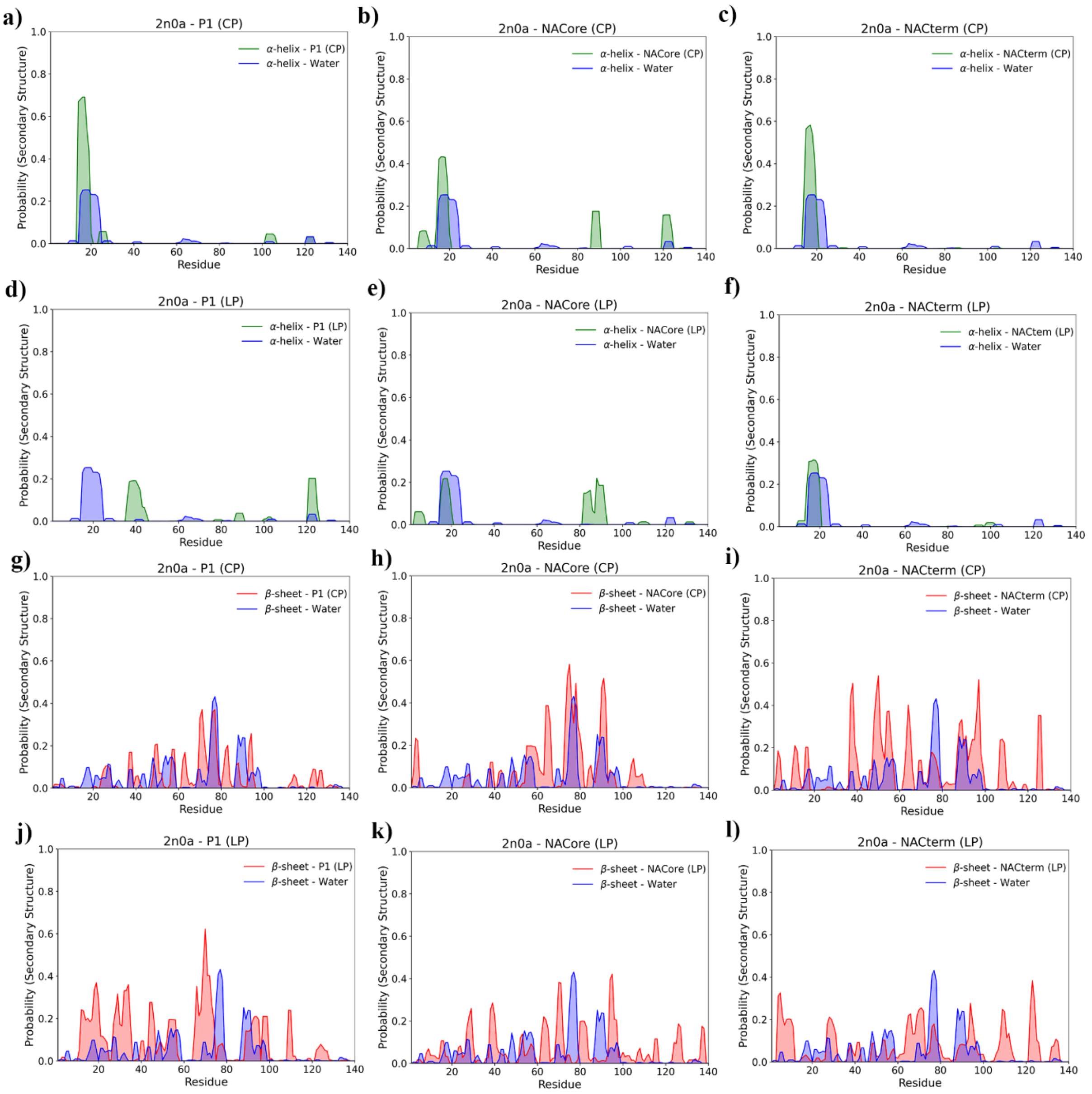

Figure S6 – α-helix **(a-f)** and β-sheet **(g-l)** distributions per residue, averaged over the last 250 ns of all trajectories, for the linear and cyclic peptides and 2n0a.

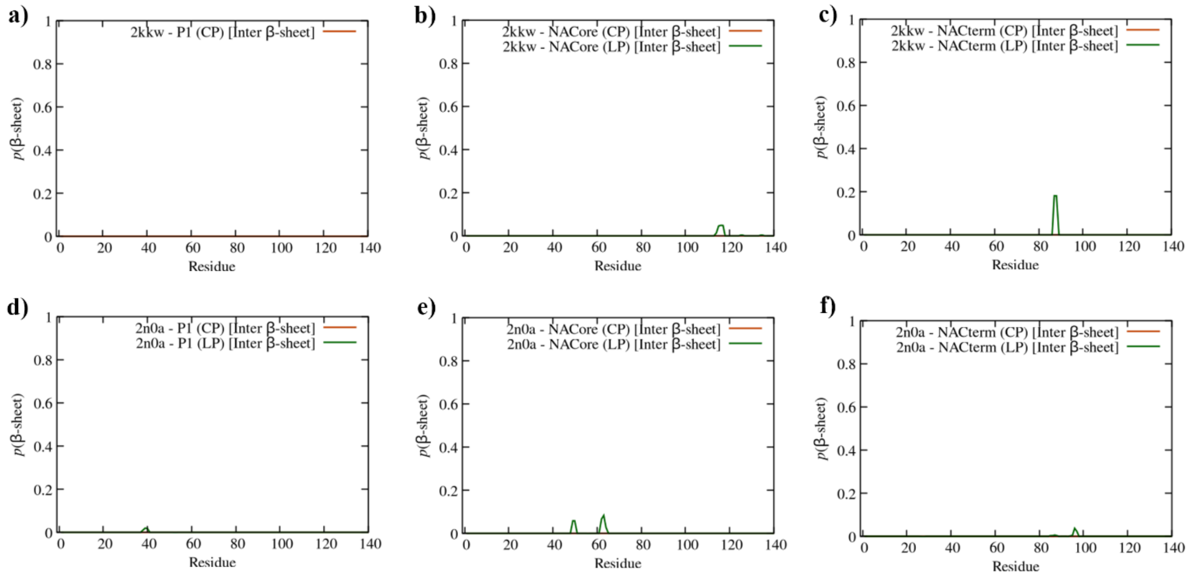

Figure S7 – Protein-peptide intermolecular β-sheet distributions per residue for the different peptides and the starting conformations 2kkw **(a-c)** and 2n0a **(d-f)**. As expected, no β-sheet is observed for the cyclic peptides. Some inter-molecular β-sheet can be seen, especially for NACore (LP) in the NAC region.
